# A Neural Network Approach to Quantify Blood Flow from Retinal OCT Intensity Time-Series Measurements

**DOI:** 10.1038/s41598-020-66158-8

**Published:** 2020-06-15

**Authors:** Boy Braaf, Sabine Donner, Néstor Uribe-Patarroyo, Brett E. Bouma, Benjamin J. Vakoc

**Affiliations:** 1 Wellman Center for Photomedicine, Massachusetts General Hospital, Boston, MA USA; 2 Harvard Medical School, Boston, MA USA; 3 Heidelberg Engineering GmbH, Heidelberg, Germany; 4 Institute for Medical Engineering and Science, Massachusetts Institute of Technology, Cambridge, MA USA

**Keywords:** Optical techniques, Imaging and sensing, Medical imaging, Retina, Biomedical engineering

## Abstract

Many diseases of the eye are associated with alterations in the retinal vasculature that are possibly preceded by undetected changes in blood flow. In this work, a robust blood flow quantification framework is presented based on optical coherence tomography (OCT) angiography imaging and deep learning. The analysis used a forward signal model to simulate OCT blood flow data for training of a neural network (NN). The NN was combined with pre- and post-processing steps to create an analysis framework for measuring flow rates from individual blood vessels. The framework’s accuracy was validated using both blood flow phantoms and human subject imaging, and across flow speed, vessel angle, hematocrit levels, and signal-to-noise ratio. The reported flow rate of the calibrated NN framework was measured to be largely independent of vessel angle, hematocrit levels, and measurement signal-to-noise ratio. *In vivo* retinal flow rate measurements were self-consistent across vascular branch points, and approximately followed a predicted power-law dependence on the vessel diameter. The presented OCT-based NN flow rate estimation framework addresses the need for a robust, deployable, and label-free quantitative retinal blood flow mapping technique.

## Introduction

Many retinal diseases are associated with abnormalities in perfusion with primary examples including age-related macular degeneration, diabetic retinopathy, and glaucoma^1–6^. Much of our current understanding in this area is derived from fluorescence angiography (FA) and, more recently, optical coherence tomography (OCT) angiography. These tools provide the morphology of the retinal vasculature, e.g., vessel diameter and capillary drop-out, but do not quantify retinal blood flow directly.

OCT imaging is commonplace in ophthalmology and an OCT-based flow imaging technique could therefore be rapidly adopted in research and clinical settings. This is especially true if the technique can be compatible with the design of commercial OCT platforms. Traditionally, flow measurements in OCT have been based on Doppler (phase-based) techniques, which measure the axial component of the blood flow^7–13^. The primary barrier to the use of Doppler OCT in the retina arises from the need to calculate total flow from axial flow using a scale factor that is inversely related to the cosine of the angle between the flow vector and the imaging beam (*i.e*. Doppler angle *α*). With *α* near 90°, a small error in the measurement of *α* leads to large errors in the measured total flow^14^. This has limited Doppler techniques to a small region near the optic nerve head where vessels are oriented to avoid *α* = 90°^15^. Across most of the retina, where vessels are oriented with *α* ~90°, only techniques such as multi-beam Doppler OCT have been successful in accurately measuring flow^16–19^. However, the use of multiple, non-colinear beams in three-dimensional imaging significantly increases the hardware complexity. The required modifications to microscope and OCT hardware are barriers in this case to a broader adoption of the technique.

The measurement instability at *α* ~ 90° for Doppler OCT is a consequence of using the OCT phase signal. To work around this problem, methods to quantify flow based entirely on the OCT intensity (or equivalently, amplitude) component have been explored^20–23^. However, the use of intensity brings its own set of challenges. First, flow stochastically modulates OCT intensity^24,25^, and it is not straightforward to estimate flow velocity from a time-series OCT intensity measurement. This is especially true when the number of measurements in the time-series is minimized^21^, as is critical in retinal imaging as the overall imaging duration is limited by eye motion^26^. Second, other effects such as Brownian motion^27^, multiple-scatting^28^, and intravoxel flow velocity gradients^29^ affect the time-series intensity modulation and further complicate the extraction of accurate flow information.

In this work, we demonstrate for the first time robust blood flow rate estimation from OCT intensity time-series measurements using a neural network (NN) analysis. We used a simple forward OCT signal model of translating scatterers to train a NN, which was used to estimate flow rates from experimental OCT signals. The NN was combined with pre- and post-processing steps to define a complete analysis framework for measuring flow rates in individual retinal blood vessels. We validated the framework’s accuracy in a blood flow phantom across flow speed, vessel angle, hematocrit levels, and signal-to-noise ratio in which Doppler OCT served as a ground truth. The framework was further validated in human retina measurements *in vivo* by confirming the conservation of flow rate across vessel branch points, and by confirming a prior reported power-law relationship between flow rate and blood vessel diameter.

## Methods

In this section, we describe first the OCT imaging system, the blood flow phantom and the scan pattern protocol used in this study. Next, we describe the NN-based and Doppler-based quantitative OCT flow methods. Finally, we describe the calculation of flow rate from flow velocity.

### Experimental setup description

#### OCT imaging system

The NN framework was implemented in OCT based on a polarization-sensitive optical frequency domain imaging (OFDI) architecture as previously described by Braaf *et al*.^30^. In short, the light from a 1-μm wavelength swept laser (100 kHz, Axsun Tech., MA, USA) was coupled into a single-mode fiber-based interferometer with a passive polarization-component depth-multiplexed design^31,32^. In the sample arm, the light was split equally by a 50/50 coupler and each output travelled a different path length through air before being recombined in a fiber-based polarization beam splitter (PBS) to generate two orthogonally polarized depth-multiplexed input states. An 80/20 coupler sent 20% of the light to the ophthalmic interface for imaging while returning light was directed to a polarization-diverse detection circuit. Here, the sample light was recombined with the reference arm, and interference was recorded separately for the horizontal and vertical polarization components. The ophthalmic interface was provided by a commercial Heidelberg Engineering Spectralis OCT device that was equipped with optics in the 1-μm wavelength range and combined the OFDI with the Spectralis scanning laser ophthalmoscope (SLO). The SLO used a 780 nm laser diode and a set of galvanometer scanners for high-speed raster scanning independent from the OFDI beam scanning. The SLO was operated at an 8.8 Hz frame rate for a 30° field-of-view from which lateral eye motions were analyzed in real-time. The obtained eye motion was converted into a correction signal and imported into the OFDI acquisition control software for on-the-fly correction of the OFDI galvanometer waveforms. The OFDI optical power on the cornea was 1.6 mW and the OFDI resolution in the axial and lateral dimensions were 10 μm (FWHM in air) and 18 μm (e^−2^ diameter) respectively. Note that the NN analysis is not dependent on a polarization-sensitive OCT architecture and can be implemented similarly on conventional non-polarization-sensitive OCT systems. For simplicity, in this study only the OFDI polarization channel with the highest SNR was used as the input for the NN analysis.

#### Blood flow phantom

**Figure 1:**
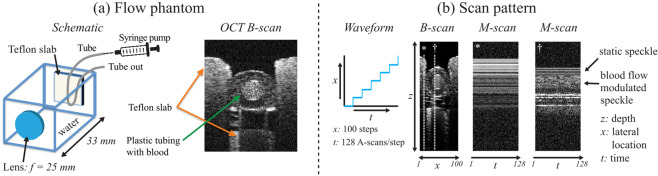
Schematic representations of the flow phantom and the scan pattern. (**a**) The phantom consisted of a water-filled plastic container with a Teflon “retina” at the back. A plastic tubing provided an artificial blood vessel that was infused with swine blood. (**b**) A stepped M-scan scan protocol was used to obtain the experimental OCT time-series data. In this protocol 128 successive A-scans were acquired from each location before proceeding to the next A-scan location.

For the purpose of calibrating and validating flow measurements, a flow phantom setup that could be interfaced to the ophthalmic microscope was developed. The flow phantom is schematically shown on the left of Fig. 1(a). This flow phantom consisted of a water-filled plastic container with a lens on the front side as the focusing optics. The size and focal length (33 mm) of the flow phantom was roughly twice that of a human eye for convenience. At the back of the phantom, a Teflon slab acted as homogenous scatterer and held a low-density polyethylene plastic tubing (Scientific Commodities Inc., AZ, USA) with 125 μm inner diameter within a groove. A syringe pump (Pump 11 Elite, Harvard Apparatus, MA, USA) was used to infuse the tubing with swine whole blood (Lampire Biological Laboratories Inc., PA, USA; CPD anticoagulant) at controlled flow rates. In the OCT B-scan on the right side of Fig. 1(a), the blood-filled tubing is shown within the groove of the Teflon slab. Compared to the human eye, the phantom’s Teflon ‘retina’ was flat, which allowed for a controlled change in the angle of incidence of the OCT beam on the tubing by changing the scan angle with which the OCT beam entered the phantom. The exact Doppler angle could be calculated from the known geometry of the phantom and the imaging scan angle. It was therefore possible to precisely measure *α* and calculate accurate flow speeds using Doppler OCT for *α* outside of an approximately 88–92° window. This allowed Doppler OCT to serve as the ground truth in the phantom experiments.

#### Scan pattern protocol

In order to obtain the experimental OCT time-series data, a stepped M-scan scan pattern protocol was used as shown in Fig. 1(b) similar to the scan pattern protocol that was proposed by Fingler *et al*.^33^ for phase-variance OCT imaging. The stepped M-scan scan pattern obtained 128 A-scans at every sample location with a parked beam before proceeding to the neighboring A-scan location. Each stepped M-scan B-scan spanned ~300 μm in width with 100 A-scan locations (and 128 A-scans at each location). The plane of the B-scan was set to be orthogonal to the tube/flow axis. The acquisition time for a single stepped M-scan B-scan was 0.13 s, and unless otherwise stated 10 repeated B-scans were obtained for each vessel location with a total acquisition time of 1.3 s.

OCT intensity images were obtained from the B-scan data by averaging the intensity information for every M-scan (see Fig. 1(b)). In the M-scan intensity images of Fig. 1(b), the time-series data obtained from static structures showed constant signals over time (denoted by *), while the speckle signals obtained from blood flow within the tubing showed rapid modulations across time (denoted by †). This clearly demonstrates the speckle intensity modulations caused by flowing blood that are quantitatively analyzed in the next sections.

### Quantitative OCT flow analysis methods

#### Neural network analysis of OCT intensity time-series datasets

We developed a NN that takes as input a measured time-series OCT intensity dataset of a prescribed length and outputs a flow velocity likelihood curve. This NN was applied pixel-by-pixel to the stepped M-scan B-scan images to generate likelihood curves for each pixel individually without including knowledge from neighboring pixels.

**Figure 2:**
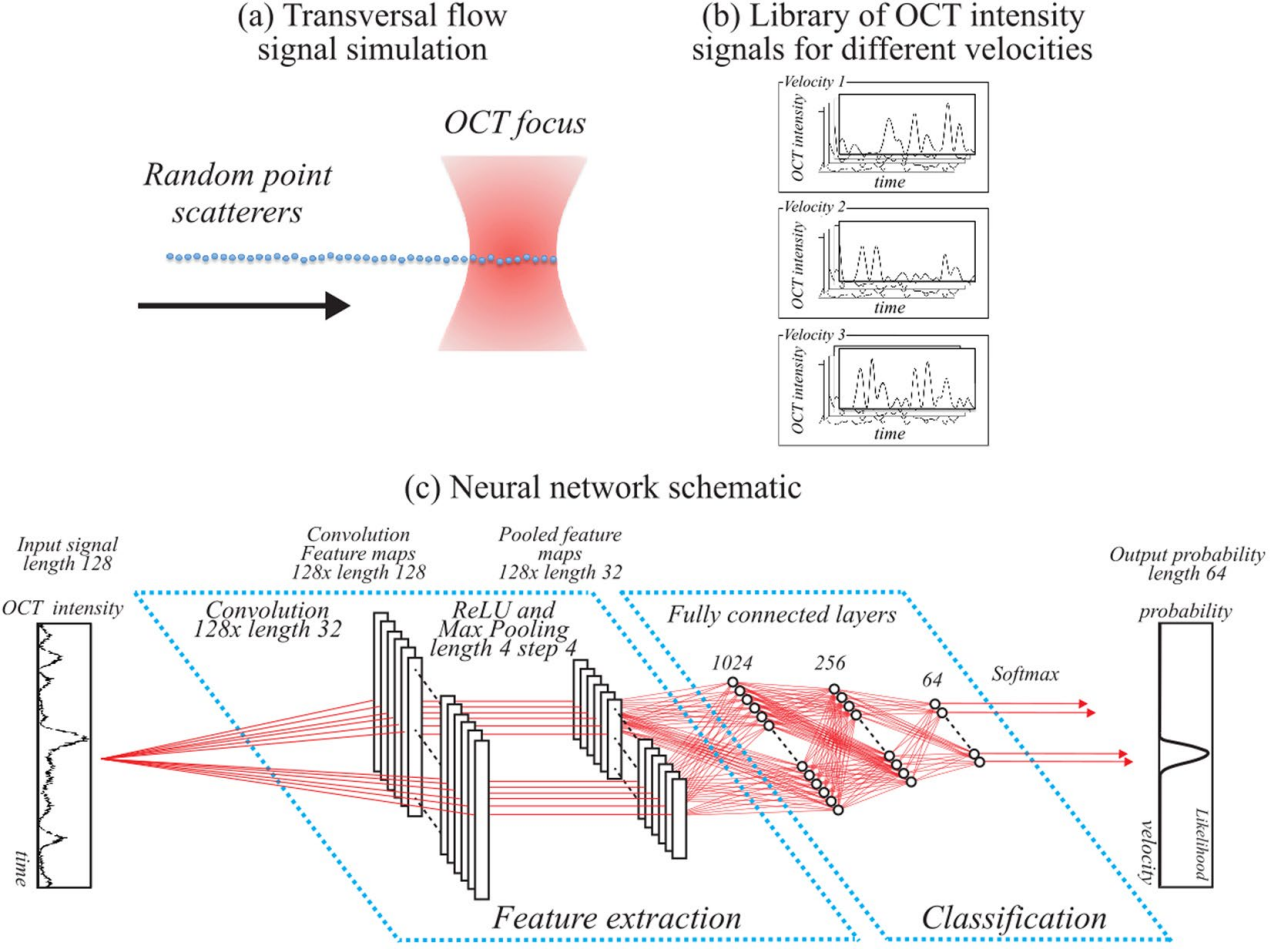
Schematic figure of the NN analysis. (**a**) OCT intensity time-series data were simulated using a forward model of transversal flow. (**b**) A signal library of temporal OCT intensity signals at different velocities was composed. (**c**) A convolutional NN was configured (see text) using a single convolutional layer, and ReLU and pooling layers for signal feature extraction; three fully connected layers were used afterwards for classification. The NN was trained using the library signals and velocity classification labels as the inputs. Experimental OCT data was analyzed by the trained NN and provided the probability likelihood that a signal belongs to a certain velocity.

In order to train the NN, we simulated OCT intensity signals generated by a simple forward model based on one-dimensional transverse flow as described previously by Vakoc *et al*.^21,24^. In short, the flow model simulates a series of randomized point scatterers that move transversely through the focus of a Gaussian OCT beam as shown in Fig. 2(a). By repeating this process with a new set of scatterer positions and scattering amplitudes, additional signal realizations for a given velocity were constructed. In accordance with Vakoc *et al*.^24^ a shot noise model was used to add noise to the signal simulation. OCT time-series signals were simulated to create a signal library for 64 different velocities with logarithmic velocity spacing between 0.001 mm/s and 2 m/s (Fig. 2b). For every velocity 25,000 signal realizations were obtained. These signals were divided into training (22,000) and validation (3,000) groups.

Each simulated and experimental time-series intensity signal had 128 time samples in accordance to the scan pattern protocol and was self-normalized to have unity mean (averaged over its 128 elements) before being used by the NN. We note for clarity that this self-normalization was applied to each time-series signal in isolation (i.e., based only on the 128 elements in its time-series), rather than using the mean calculated over a broader set of signals.

A convolutional NN was configured and empirically optimized using the Neural Network Toolbox in Matlab 2017b (The Mathworks, Inc., MA, USA) (Fig. 2c). The network structure was configured to process a single time-series intensity signal with 128 points. Signal feature extraction was performed from the input signal by a single convolutional layer with 128 one-dimensional kernels with a length of 32 samples. A ReLU layer was included after this convolutional layer to suppress negative network activation and a Max Pooling layer of length 4 and step 4 was used to decrease the data size after the feature extraction. Successively, signal classification was performed by three cascaded fully connected layers with 1024, 256 and 64 nodes. The output of the NN was given by the softmax function, which generated a likelihood function that described the probability that the input signal belonged to any of the 64 classification velocities. The network was trained for 200 Epochs in which its velocity classification was optimized to best match the velocity classification labels. We note that we did not rigorously optimize NN architecture, nor did we attempt to reduce NN size for efficiency goals. The focus of this work was on demonstrating performance in a NN-based approach. As such, it is likely that directed efforts may provide further gains in performance and/or computational efficiency.

In our preliminary testing of the NN, we observed a relatively strong dependence on measurement SNR. For example, both simulated and empirical signals with low or moderate SNR were poorly classified by NNs trained on noise-free simulated signals. This SNR-bias was mitigated by training a set of distinct NNs across a range of SNRs (5 to 35 dB with 1 dB increments). Then, when analyzing a given time-series, the SNR of the measured signal was estimated and an appropriate NN was selected. Separate training and validation signals were generated for each of the 64 velocities, and within each velocity for each of the 31 SNRs. This yielded a total of 49.6 million (64 × 31 × 25,000) time-series datasets.

Experimental OCT data was analyzed with the trained NN which determined the likelihood function that a specific signal belongs to a certain velocity. Spatial filtering was applied on the OCT B-scan level for further noise suppression using a 3 × 3 pixel kernel that elementwise multiplied the likelihood functions of the included pixels. The maximum of the filtered likelihood function provided the velocity classification for each individual B-scan pixel.

#### Doppler OCT analysis

OCT phase information was used to obtain bi-directional Doppler flow images derived from the phase-difference between A-scans^11,12^. The flow velocity was assumed constant for the obtained 128-point OCT time-series dataset. This allowed for the calculation of phase-difference images between different time points within one time-series dataset that could be averaged to suppress noise. In addition, multiple different time delays could be used for the phase-difference calculation, which were all scaled to a time delay of 10 μs (1 A-scan) and averaged to further suppress noise. In this study, phase-difference images were calculated for time delays ranging from 10 μs (1 A-scan) up to 400 μs (40 A-scans) in steps of 10 μs (1 A-scan). This generated respectively 127 (1 A-scan) to 88 (40 A-scans) phase-difference images per time delay with a combined total of 4300 phase-difference images over all time delays. In order to average all these phase-difference images together for the best noise suppression, first the phase-difference images were averaged per time delay individually. These averaged phase-difference images per time delay hold the same velocity information but are scaled according to their time delays. However, phase wrapping, bulk motion and phase decorrelation effects can manifest differently for each time delay and should therefore be addressed before further averaging is applied. Phase wrapping was compensated using a two-dimensional phase-unwrapping algorithm^34^ and bulk motion was compensated by subtracting the average phase from each A-scan pair obtained from static pixels with significant SNR (>3 dB)^12^. The discontinuous phase points that resulted from (i) noise, (ii) phase decorrelation and/or (iii) unwrapping errors were detected^34^. These affected phase-difference images were excluded from further analysis. The remaining phase-difference images were scaled to a time delay of 1 A-scan and averaged. The final averaged phase-difference image was then used to calculate cross-sectional velocity images using *v*_*flow*_ = (Δ*ϕ*·*λ*_0_)/(4π·*n*·*τ*·cos(*α*)), where *v*_*flow*_ is the flow velocity, Δ*ϕ* is the observed phase-difference, *λ*_0_ is the center wavelength of the light source (1040 nm), *n* is the refractive index of blood (1.36), *τ* is the time delay between the two A-scans, and *α* is the Doppler angle.

#### Calculation of flow rate (µL/min) from flow speed (mm/s)

The cross-sectional velocity maps from the NN and Doppler methods were used to calculate the flow rate of (artificial) blood vessels. The flow rate of every pixel in the cross-sectional map was calculated as *f*_*rate*_ = *v*_*flow*_ * *A*_*pix*_, with *f*_*rate*_ as the flow rate, *v*_*flow*_ as the flow velocity and *A*_*pix*_ as the cross-sectional area of a pixel. *A*_*pix*_ was obtained by multiplying the lateral step size between neighboring A-scan locations in the stepped M-scan B-scan and the axial OCT pixel size scaled for the refractive index of blood (*n*_blood_ = 1.36). The flow rate for the blood vessels would be determined by integration of the flow rate over the pixels inside the lumen of the blood vessel. For this purpose, the lumen of the vessels was segmented manually for the flow phantom tubing or by OCT angiography image processing for the *in vivo* vessels. The cross-sectional luminal area considered in the flow rate calculation was dependent on the experiment as described below. The flow rate was converted for graphical display from μL/s to μL/min by multiplication with 60 s/min.

### Human subject imaging

The measurements of human retinas *in vivo* adhered to the tenets of the Declaration of Helsinki and were approved by the Massachusetts General Hospital Institutional Review Board. Informed consent was obtained from the imaged subject.

## Results

In the result section the dependence of the NN analysis on Doppler angle α is described, and a method that mitigates this dependency is presented. Successively, the necessary linear scaling of NN estimated flow rate to obtain correct values is analyzed, as well as its dependency on measurement SNR and hematocrit level. In addition, the NN analysis was validated with *in vivo* retinal measurements of flow rate preservation across vessel bifurcations and for the flow rate dependency on blood vessel size.

### NN flow rate estimation depends on Doppler angle

**Figure 3:**
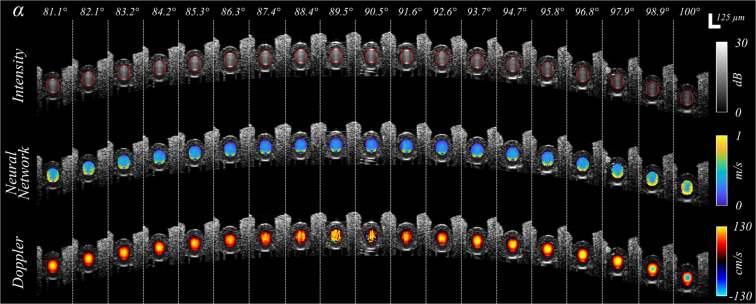
NN flow velocity estimation is stable across perpendicular Doppler angles but suffers from edge artifacts that increase as α deviates from 90°. The first row presents intensity images of the structure of the tubing, with the segmentation of the tubing lumen denoted in red. The second row presents the NN velocimetry data inside the tube overlaid on the intensity images. The third row shows the Doppler OCT velocimetry data (total flow), again overlaid on the intensity images. Each column as denoted by the white dashed lines represents a distinct measurement at different locations and Doppler angles (as labeled) in the same tube. Swine blood was infused at a fixed flow rate of 50 µL/min for all measurements.

**Figure 4:**
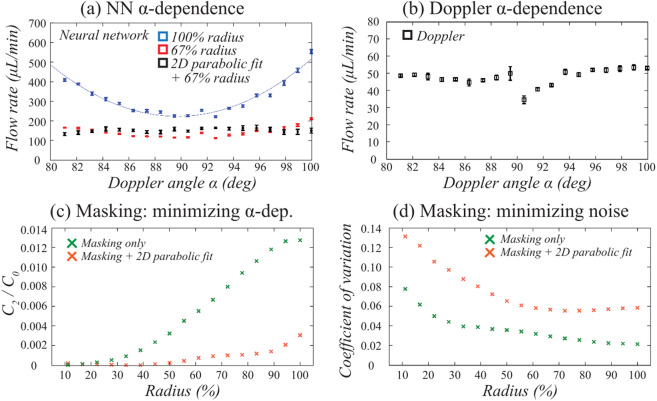
Doppler angle influences both NN and Doppler methods, but its impact on NN estimations can be minimized by spatial masking. (**a**) NN estimated flow rates calculated by summing flow speeds across the full tube aperture (blue) and by restricting the calculation to use an aperture radius (Ra) equal to 67% of the tube radius (red). A two-dimensional parabolic fit to the restricted aperture data further reduced the dependency on Doppler angle (black). The fit in blue to the full tube aperture data describes a parabola, y = Co + C_1_(α − 90°) + C_2_(α − 90°)^2^, from which the *C*_0_ and *C*_2_ coefficients are used for the optimization of the masking. (**b**) The Doppler OCT total flow measurements across Doppler angle show expected instability near 90°. (**c**) NN flow rate estimates were calculated as a function of the applied spatial masking aperture, Ra. For each value of Ra, the NN flow rate as a function of α was fit to a parabola. The value of C_2_/C_0_ was plotted as a function of the aperture radius. (d) The influence of using reduced apertures, Ra, on the measurement variability was quantified by calculating the coefficient of variation as a function of aperture radius.

To characterize the influence of Doppler angle on the accuracy of NN flow rate estimation, we measured the flow phantom at 19 distinct locations, each with a distinct Doppler angle across a range from 80° to 100°. In order to facilitate the visual comparison between the NN and the Doppler OCT results, the latter was corrected for the Doppler angle and displays total (*i.e*. not axial) flow. In Fig. 3, cross-sectional intensity and velocimetry data (NN and Doppler OCT) are shown for each Doppler angle, while Fig. 4 shows quantitative flow rate results. Figure 3 shows Doppler OCT images without phase unwrapping or the rejection of noise affected images to clearly demonstrate the challenges for this method. The results highlight several important points. First, the NN flow velocity estimation is stable throughout the full angular range, *i.e*., the NN method does not suffer from instability at α = 90°. However, there are artifacts at the lateral and bottom edges of the tubing for the NN method. These artifacts at the bottom of the tubing are apparent for all Doppler angles, and their origin is not fully understood at this time. The artifacts at the lateral edges of the tube grow as |α − 90°| increases, which can be clearly observed from the quantitative results in blue in Fig. 4a. These are consistent with the predicted decorrelation effect of velocity gradients in the axial velocity within the measurement voxel^29^. Second, the Doppler OCT measurements show the predicted instability for α sufficiently near to 90°, while providing precise flow rates for Doppler angles outside the 88–92° window (Fig. 4b). For α > 98°, flow profile discontinuities due to phase wrapping were observed (Fig. 3). We note that the phantom setup afforded a more precise measurement of Doppler angle than is possible *in vivo*, and thus these Doppler measures primarily serve to provide a ground truth upon which to validate the NN results.

### Spatial masking reduces the dependence of the NN flow rate estimation on Doppler angle

The increase in the NN flow rate estimation for larger values of |α − 90°| as shown in blue in Fig. 4a can be attributed to the artifacts at the tubing wall. Excluding the outer most regions of the tubing cross-section in the flow rate determination should therefore diminish this effect. We therefore calculated the flow rate using a spatial masking approach wherein the pixels within a radius Ra from the tube center are used, and Ra is defined as percentage of the tube radius (*i.e*., Ra = 100% implies no aperture). The NN flow rate estimates when using masking with Ra = 67% showed dramatically reduced dependence on α (Fig. 4a, red datapoints). The selection of Ra = 67% was motivated by the competing goals of reducing α dependency through smaller Ra, and reducing measurement variability through use of more measurements (pixels) and therefore a larger Ra. These dependencies are plotted in Fig. 4c,d. To extend the flow measurements to the excluded regions of the tubing, the data from within the aperture was fit with a 2D parabola that was forced to zero velocity at the tubing wall. This further reduced the Doppler angle dependency as shown in black datapoints in Fig. 4a. In Fig. 4c one can appreciate that spatial masking and parabolic fitting significantly reduces the Doppler angle dependency from the reduction in the scaled quadratic fit coefficient. In Fig. 4d it is shown that masking and parabolic fitting reduced the amount of included data (pixels) and therefore increased the measurement variability. At Ra = 67% a fair compromise between the two effects was found. Thus, the edge effects, both at the lateral and bottom edges, can be effectively removed through modest spatial masking. Unless otherwise noted, flow rate measurements integrated across the tube diameter in this work were calculated using spatial masking with Ra = 67%.

### NN flow rate estimation is accurate to within a single scale-factor

**Figure 5:**
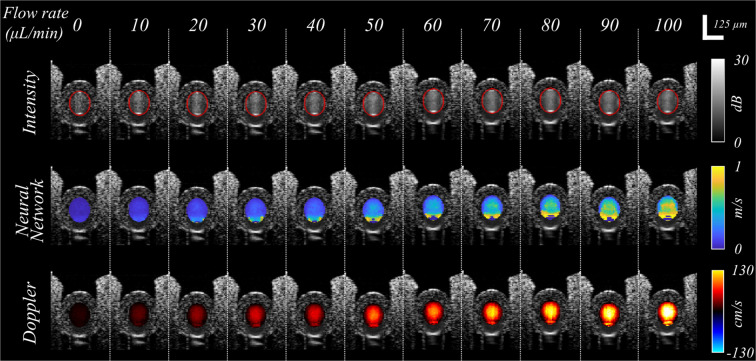
The NN and Doppler OCT measurements with varying flow rate. The first row shows intensity images of the structures of the tubing. The second row shows the NN velocimetry data from inside the tubing as an overlay on the intensity images. The third row shows the Doppler OCT velocimetry data as an overlay on the intensity images. Each column as denoted by the white dashed lines represents a different tubing infusion flow rate.

**Figure 6:**
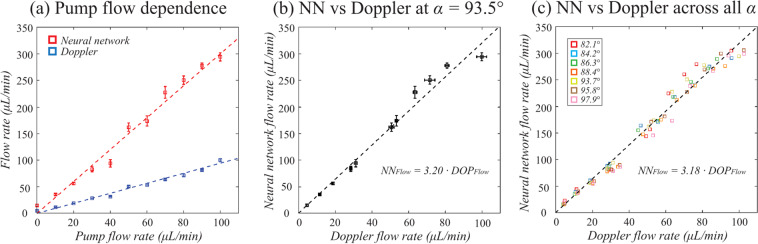
The relationship between NN and Doppler measured flow rates. (**a**) The detected flow rates are plotted as a function of the set pump flow rate for an angle of incidence of 93.5°. Each datapoint shows the average and standard deviation of the measured flow across 10 repeated measurements. (**b**) The NN flow rate is plotted versus Doppler flow rate for an angle of incidence of 93.5°, revealing a 3.2 scale-factor difference between NN and Doppler (ground truth). (**c**) Across all angles, the scale factor relating NN to Doppler (ground truth) was calculated to be 3.18. Note that measurements with Doppler angles close to 90° were excluded from this calculation due to high error/noise in the Doppler technique.

To evaluate the accuracy of the NN estimation, measurements were acquired across Doppler angles from 80° to 100° and across pump flow rates up to 100 µL/min (277 mm/sec peak speed at tube diameter of 125 µm). In Fig. 5, cross-sectional intensity and velocimetry data are shown at α = 93.5°. Similar to Fig. 3, the first row shows intensity images, and the second and third rows show the velocimetry images of the NN and Doppler OCT methods, respectively. The Doppler OCT data was corrected for its Doppler angle in order to show the total (*i.e*., not axial) flow rate. Each column defined by dashed white lines shows data from different flow rates, for which the flow rate labels are given by the infusion pump. When total flow rate was extracted using the spatial masking approach described above, the results of both the NN and Doppler methods scaled in proportion to the pump’s set flow rate (Fig. 6a). However, while Doppler OCT matched the pump setting, the NN method deviated from the pump flow rate by a fixed multiplicative factor. Using the Doppler measurements as the ground truth, this multiplicative factor was measured to be 3.2 (NN to Doppler) at α = 93.5° (Fig. 6b). The scale-factor difference is likely due to a combination of Brownian motion, multiple-scattering, and residual flow velocity gradient effects. Next, we confirmed that the scale factor was largely independent of Doppler angle and derived a single scaling factor of 3.18 across all Doppler angles (Fig. 6c). Hereafter, all measured NN speeds and flow rates are reduced by this empirically derived fixed scale factor.

### NN flow rate estimation is minimally biased by measurement SNR

**Figure 7:**
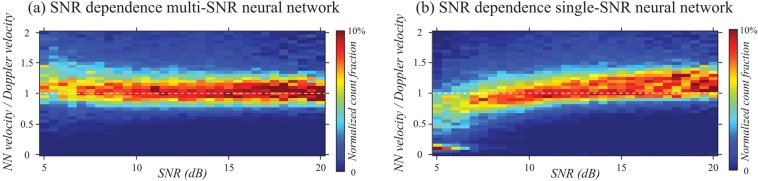
Using SNR-dependent NNs significantly reduces the SNR-bias in flow rate estimation. (**a**) Using SNR-dependent NNs, the distribution of the ratio of NN to Doppler flow velocity estimates are centered at unity for measurements SNRs from 5–20 dB. (**b**) Using a single NN trained at an SNR of 15 dB, the distribution shows a pronounced SNR bias.

To characterize the extent that measurement SNR biased the NN flow speed estimate, the NN and Doppler OCT flow speeds were compared at each pixel within the 67% masking radius aperture region (Fig. 4). The ratio between the NN and Doppler flow speed was analyzed as a function of the measurement SNR which varied across depth and Doppler angle, the latter due to a slight defocusing effect. We note here that noise does not induce a significant bias in the Doppler OCT (mean) flow velocity estimate for a large set of measurements^35^. The flow phantom tubing was infused with swine blood at a 30 μL/min flow rate and data was acquired for Doppler angles between 2° and 10° away from perpendicular angle of incidence. Measurements were binned according to SNR with 0.5 dB bin spacing and the histograms of NN to Doppler estimates for each bin (self-normalized within the bin) were calculated (Fig. 7). In Fig. 7(a), the results are shown using distinct NNs for each measured SNR. The great majority of the measurements with SNRs between 5 and 20 dB have a velocity ratio that is close to 1, which indicates negligible differences in the velocimetry data between Doppler OCT and the NN. For comparison, Fig. 7(b) shows a similar analysis using a single NN trained only with signals at an SNR of 15 dB. Here, an SNR bias is observed with the NN overestimating and underestimating flow velocities when SNRs are above and below the 15 dB training SNR value, respectively. These results demonstrate that the NN flow rate estimation is minimally biased by measurement SNR when the NN analysis is trained across the appropriate experimental SNR range.

### NN flow rate estimation varies by less than ±4.4% across the physiological hematocrit range

**Figure 8:**
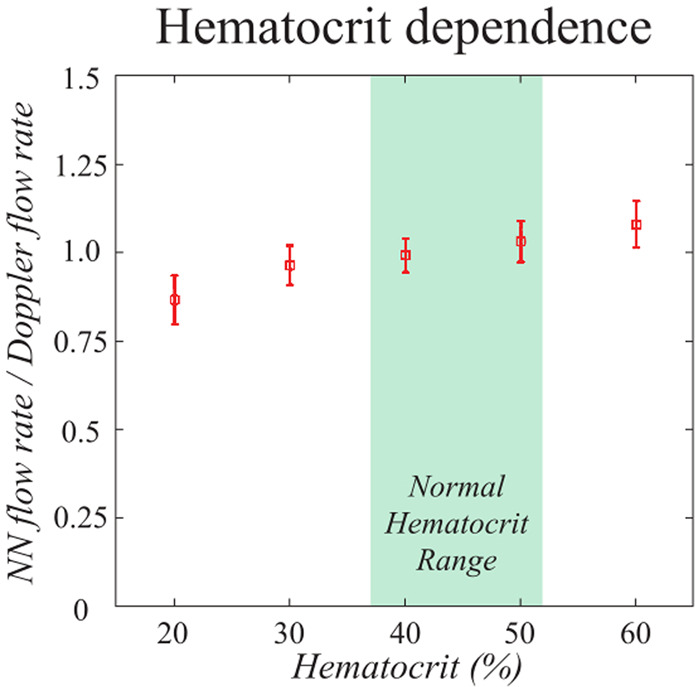
Physiological variations in hematocrit have a limited effect on NN flow rate estimates. The physiological range of human hematocrit is shown in green (37–52%). Increasing hematocrit resulted in slight increases in estimated flow rates, but these effects are likely to be dominated by other sources of *in vivo* measurement variability. The data points show the average and standard deviation of the estimated flow rate over all Doppler angles greater than 2° from perpendicular.

Hematocrit levels vary typically between 37–52% across individuals^36^. To estimate the impact of hematocrit levels on NN flow rate estimates, we performed measurements in the flow phantom with a series of dilutions to swine blood samples. Swine blood was stored in tubes for over four hours to let the blood cells settle and separate from the blood plasma. Plasma was added or removed from the samples to vary the hematocrit level from 20 to 60%. Afterwards, the samples were homogenized by mixing. The flow phantom tubing was infused with the blood samples at an infusion flow rate of 30 μL/min flow rate and data was acquired for Doppler angles outside of 88–92°. As in the prior validation experiments of the SNR dependence in the previous section, we compared NN estimates to Doppler estimates. Doppler measurements are known to have negligible dependence on hematocrit^37^. In addition, by comparing NN and Doppler OCT directly within the same measurement, we control for unavoidable variations in achieved pump speeds. We observed that NN flow rate estimates increase slightly as hematocrit increases (Fig. 8). Within the physiological range of 37–52%, NN flow rates varied by ±4.4%. This variation is on the same order as those induced by measurement variability (error bars). These results suggest that physiological hematocrit variations have limited influence on blood flow rate estimates using NNs, and that it is reasonable to neglect hematocrit dependence in most applications.

### NN flow rate estimates are conserved across branch points in the human retina

**Figure 9:**
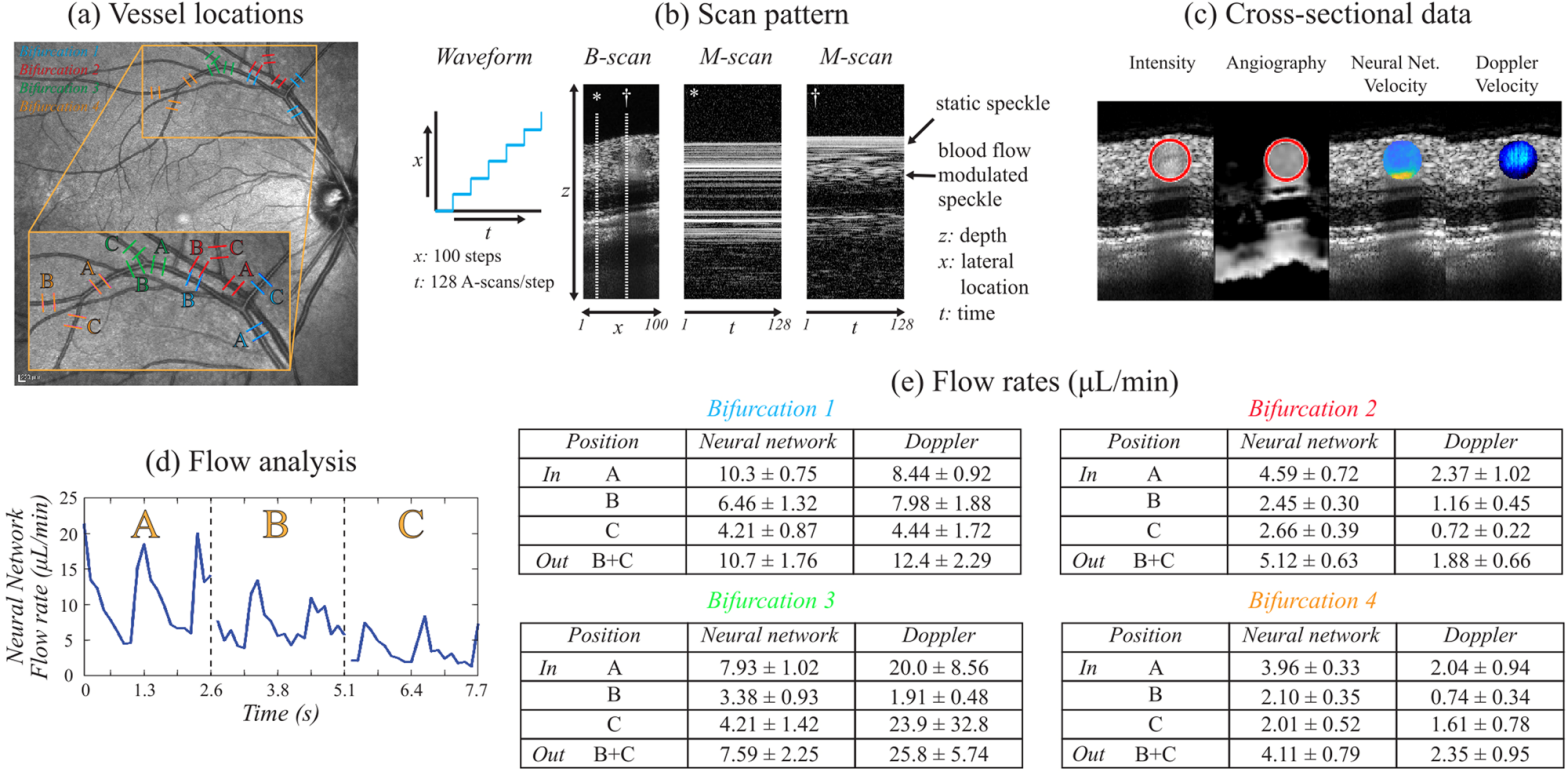
Scan protocol and results of *in vivo* NN flow rate estimation across bifurcations. (**a**) Four vessel bifurcation locations are indicated with their inflow (A) and outflow (B,C) vessel segments. Bifurcations 1–4 are illustrated in blue, red, green, and orange, respectively. (**b**) The stepped M-scan scan pattern protocol that was used for data acquisition. Regions of flow and static tissue can be identified based on the visibility of the speckle features (B-scan view) or the temporal modulation of intensity (M-scan views). **(c**) At each measurement location cross-sectional images were obtained for the intensity, angiography, NN velocity and Doppler OCT velocity. (**d**) In the artery measured at bifurcation 1 clear cardiac cycle signals could be measured with the NN analysis. (**e**) The average ± standard deviation of the flow rates measured for each vessel segment across 10 datasets for each bifurcation. NN estimated flow rate is conserved across bifurcations.

To demonstrate that the NN approach can be successfully applied to the human retina, and to provide a preliminary validation of the NN estimated flow rates *in vivo*, we designed a scan pattern to measure the total inflow and outflow across vessel bifurcation points in a healthy volunteer similar to the approach of Trasischker *et al*.^18^. Four vessel bifurcations were selected from the SLO retinal *en face* image as shown in Fig. 9(a). Each bifurcation was imaged at its three vessel segments (indicated as A, B, C), and each vessel segment was imaged at two locations (*e.g*., A1 and A2) spaced by 200 µm to allow Doppler angle calculation (Fig. 9a). A stepped M-scan scan imaging protocol was used as shown in Fig. 9(b) which is similar as the one described in the methods section. The imaging protocol was as follows: 20 M-scan B-scans were acquired in an alternating fashion at locations A1 and A2 for bifurcation 1 (*i.e*., A1, A2, A1, A2, …). This pattern was then repeated at locations B1 and B2 for bifurcation 1, and then C1 and C2 for bifurcation 1, and again across the remaining bifurcations 2–4. In post-processing, the vessel lumen was detected from the OCT angiography image as shown in Fig. 9(c) and velocimetry data was obtained with both the NN and Doppler OCT analyses. In contrast to the phantom eye blood flow, the *in vivo* eye showed clear cardiac cycle pulsation as is shown in Fig. 9(d) for the first bifurcation as measured by the NN analysis. Vessel segment A provided the inflow and thus showed stronger flow rates than the outflow from vessel segments B and C. In the Doppler OCT analysis the Doppler angle was estimated from the shift in depth position of the blood vessel between the two imaging locations for each vessel segment similar as described by Makita *et al*.^38^. Measurements with Doppler angles from 88° to 92° were rejected due to their lack of reliability. Finally, the average flow rate across a cardiac cycle per vessel segment was calculated using both NN and Doppler methods.

The table of Fig. 9(e) provides the mean and standard deviation of the measured flow rates across 10 paired datasets (for each location) for each vessel segment A-C. Also shown is the total outflow calculated by adding the flow of segments B and C. The NN estimated inflow (A) and outflow (B + C) are in good agreement for all four vessel bifurcations. In contrast, the Doppler inflow and outflow measurements are more discordant. Due to the steep Doppler angles encountered for the four bifurcations 54% of the obtained scans were rejected from the Doppler analysis. In addition, a significant Doppler angle variation for the remaining scans, which was often higher than 1°, caused large discrepancies in the Doppler OCT measured flow rates. This demonstrates the challenges of applying Doppler OCT in the retina, and the benefit afforded by intensity-based approaches like the NN framework.

### NN flow rate estimates follow known power-law dependence on vessel diameter in the human retina

**Figure 10:**
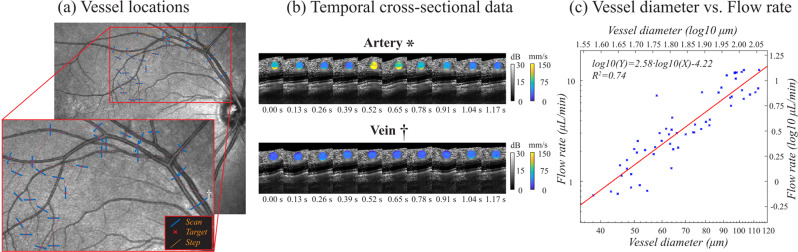
NN flow rate estimation follows known scaling with vessel diameter. (**a**) 30 vessel locations were imaged with the stepped M-scan scan protocol to measure flow rate data with the NN analysis. (**b**) For every vessel location 10 successive scans were obtained that are shown here in filmstrip format for a central artery (*) and a central vein (†). The velocimetry data is overlaid in color on the OCT intensity images. (**c**) The measured flow rates as a function of vessel diameter on logarithmic scale.

Because the vessel diameters of the inflow and outflow segments differed in the above bifurcation experiments, a significant dependence of NN flow rate estimates on vessel diameters would likely result in unequal inflow and outflow measurements, which was not observed. To further explore vessel diameter dependencies, we asked whether NN estimated flow rates follow known power-law scaling with vessel diameter^15,19,39–42^. Thirty vessel locations were selected in the *en face* retinal SLO map of a healthy volunteer. Both arteries and veins up to their 4^th^ branches order were included (Fig. 10(a)). A custom scan pattern was configured to scan each vessel location with 10 successive stepped M-scan scans (at a single location, Doppler measurements were not obtained in this experiment). This allowed for the measurement of the average flow rate across the cardiac cycle with an acquisition time of 1.3 s per vessel location and a total acquisition time of 39 s. In Fig. 10(b) the cross-sectional velocimetry data of the NN analysis is shown overlaid on the OCT intensity images for central artery (*) and vein (†) locations as indicated in Fig. 10(a). Figure 10(b) shows the successively obtained cross-sectional images in a film strip format. Here, the pulsatile flow velocity of the cardiac cycle can be clearly observed for the central artery, while more constant flow velocities were observed for the central vein. These findings are in good agreement with the known physiology of the human retinal vasculature system. The average flow rate and diameter were obtained for every vessel location. Scans that were affected by eye motion were manually rejected. The data from three different datasets were included in the analysis. The data from both arteries and veins were pooled together as prior studies found no significant difference in their power-law dependence^15,19^.

In Fig. 10(c), the measured flow rate is plotted as a function of vessel diameter on logarithmic scale. A good linear correlation (R^2^ = 0.74) was found between the two variables as denoted by a linear fit (in red). The linear fit slope of 2.58 was in good agreement with the slopes found by other OCT studies (1.97–2.52)^15,19,42^ and laser Doppler flowmetry studies (2.76–3.35)^39–41^. This result indicates that the flow rates reported by the NN analysis are consistent with the known power function scaling with vessel diameter, and this provides further evidence that vessel diameter in the range of 36 μm to 120 μm is not a significant confounding factor in the NN flow rate estimation.

## Discussion and Conclusion

In this study we described a NN approach for quantitative flow analysis in the retina. Validation and calibration was performed in a flow phantom for dependencies on angle of incidence, flow rate, SNR and hematocrit level. The NN analysis was validated *in vivo* by showing flow rate preservation for vessel bifurcations and by verifying the expected power function relation between flow rate and vessel diameter. An important aspect of the NN analysis method is that it performs well when the angle of incidence is perpendicular, which makes it robust in retinal imaging. The NN method is therefore a promising new tool for fundamental research and clinical diagnostics of retinal diseases that affect the vascular network such as age-related macular degeneration and glaucoma.

The challenges intrinsic to using Doppler methods to measure blood flow in the human retina have been well described^14^ and likewise the potential advantage of adopting intensity-based approaches to reduce angular sensitivity are broadly known^43^. However, few demonstrations of intensity-based flow quantification in blood flow phantoms exist in the literature and no demonstrations of intensity-based flow quantification (as distinct from qualitative measures such as described in^44^) in the retina have been reported. This work provides a framework for estimating flow velocity and flow rates from intensity-based time-series OCT data and demonstrates the accuracy and reliability of this approach across a broad parameter space both in phantom and in human eyes *in vivo*. However, given that there is no convenient validating method for measuring retinal blood flow, the validations *in vivo* have limitations. First, while the flow measurements are self-consistent across branch points and broadly follow known power-law scaling with vessel diameters, these are relative measures and it will be critically important in future work to employ more advanced validating methods such as fluorescence-based measurements or multi-beam Doppler OCT to provide absolute flow velocity calibration. Second, the exploration of the NN framework accuracy across (physiological) vessel diameters was limited in the phantom studies, largely due to the experimental challenges in creating flow tubing smaller than the 125 µm diameter tubes employed in these studies. Finally, the spatial masking approach used to reduce angular dependence that is driven by flow velocity gradients was shown to be effective in relatively large vessels but may have diminishing value when applied to smaller vessels that approach the resolution of the OCT system.

The goal of this work was to introduce a NN framework for estimating flow rate, and to provide validation studies in both phantoms and human eyes *in vivo*. To this end, a conventional retinal OCT system operating at moderate speeds (100 kHz) was used with uniform-in-time sampling for time-series measurements. We note that the use of faster systems with MHz speeds will likely be a critical feature in any successful deployments of retinal blood flow imaging and future work will need to focus on the adaptation of flow imaging methods to faster systems^45^. To this end, we highlight the flexibility that the NN framework allows in the timing of measured data. Non-uniform temporal sampling can realize a more efficient flow estimation across a given dynamic range^21^ and the NN method can easily support arbitrary temporal sampling strategies. This would allow for the necessary future optimization of the temporal sampling in applications that are affected by sample motion (e.g. retinal flow imaging) and for a more efficient measurement over wide flow velocity ranges in vascular systems with large vessel diameter variations.

This study analyzed the results of the NN analysis at the vessel level, not at the pixel level. The relatively large diameters of the vessels targeted allowed spatial averaging, which reduced the impact of pixel-level flow rate estimation errors. It remains unclear if this NN, or any other intensity-based approach, can reliably estimate flow from a short time-series dataset for a single pixel. Furthermore, bulk sample (eye) motion occurs during the measurements and can cause flow velocity misclassification. Care should be taken to identify and discard motion-corrupted measurements. Lastly, while the use of SNR-dependent NNs minimized noise bias in estimated flow rates, the current framework was unable to reliably estimate flow for SNRs below 4 dB for which rapid random noise fluctuations dominated and led to flow rate overestimation.

The NN performance was compared directly against Doppler measures in controlled phantoms and was evaluated for self-consistency in human eyes *in vivo*. It would be interesting to explore the performance of the NN approach relative to more conventional decorrelation methods. Such a comparison, being beyond the scope of this work, may lead to further refinements in the accuracy and efficiency of intensity-based analysis frameworks. For verification purposes, several of the developed analysis steps for the NN framework (e.g. the spatial masking) were also implemented for conventional decorrelation analysis (as described in^23^) with similar success. This emphasizes that the analysis methods described in this paper address fundamental issues of analyzing OCT intensity time-series data that are independent from the analysis tool.

As a final point, all phantom studies reported here were also performed with an intralipid scattering fluid. These data are not shown, in part because they are superseded by the blood work, and further because the results diverged significantly from those of blood. This highlights what has been previously reported – that the unique scattering properties of blood affect the performance of intensity-based flow estimation algorithms^46^ and intralipid studies are therefore of limited value in the development of *in vivo* human quantitative blood flow measurement techniques.
